# Cutting of Diamond Substrate Using Fixed Diamond Grain Saw Wire

**DOI:** 10.3390/ma15165524

**Published:** 2022-08-11

**Authors:** Osamu Kamiya, Mamoru Takahashi, Yasuyuki Miyano, Shinichi Ito, Masanobu Nakatsu, Hiroyuki Mizuma, Yuichi Iwama, Kenji Murata, Junpei Nanao, Makoto Kawano, Arata Maisawa, Takashi Kazumi

**Affiliations:** 1 Department of System Design Engineering, Faculty of Engineering Science, Akita University, Akita 010-8502, Japan; 2 SANWA TEKKI CORPORATION, 6-4-6 Minami-Shinagawa, Shinagawa-ku, Tokyo 140-8669, Japan; 3 Research & Development Department, Nippon Koki Co., Ltd., Shirakawa 961-8686, Japan

**Keywords:** diamond cutting, diamond saw wire, fixed grain saw wire, diamond grain, cutting length

## Abstract

This study demonstrates that a single-crystal diamond substrate can be cut along designed lines using the diamond-saw-wire cutting method. We developed an original saw-wire fixed diamond-grain using a bronze solder with a high melting temperature. We created a unique product machine system with a high vacuum furnace and a bronze solder that contains a metallic compound. The diamond cutting mechanism employed in this study is based on the mild wear phenomenon, owing to the friction between the diamond surfaces. A linear relationship between the cutting length and wire feed distance was observed. The relationship can be approximated as *y* = 0.3622*x*, where *y* (μm) is the cutting depth and *x* (km) is the wire feed distance. The life of the saw-wire was longer than that of the 6000 km wire feed distance and was tested by reciprocating an 8-m short wire at a speed, tension, and cutting force of 150 m/min, 1 N, and 0.2 N, respectively. A single crystal diamond substrate could be cut along the designed line, which was more than 2 mm long. The cutting speed was maintained constant at 0.36 μm/km.

## 1. Introduction

Micro-wire saw cutting is widely used in traditional manufacturing for fabricating numerous micro-electronic devices. Recently, this technique has been utilized for solar cell manufacturing. Diamond semiconductors have been regarded as power devices, because of their high thermal conductivity and electron mobility [1]. The size of industrial products has reached the two inch diameter of the diamond substrate [2] and therefore the market is expanding rapidly [3]. Generally, the diamond substrate is cut using a revolving blade [4,5], laser beam [6,7], or cleaving method [4]. The blade saw can cut the diamond along a flat surface, rather than a curved surface. Setting up a facility for laser cutting is significantly expensive and time-consuming. The cleaving method is only used for the cleavage plane of diamond crystals [8]. Thin diamond substrates can be cut by the focused ion beam (FIB) process [9].

Comparatively, saw wire cutting along curved lines with less damage is expected to be utilized for cutting diamond substrates. However, saw wire cutting is mainly used for non-diamond substrates [8,10,11]. Conventional wire sawing utilizes a slurry that contains abrasive grains in the cutting liquid, which causes environmental pollution. Recently, an ecofriendly fixed grain-type-saw-wire has been developed, to increase the cutting speed [12]. There are three methods to fix the grain-saw-wire via the grains bonds: using nickel plating [13], polymer adhesive [14], and metal solders [15]. The strength of diamond grains fabricated with metal solder is higher than those fabricated employing the other methods. Ishikawa presented a diamond grain fixed using a metal solder-saw-wire. In this research, the shearing strength of the bronze metal solder was approximately 800 MPa [16], and the diamond grain was strongly fixed. Therefore, the metal solder-saw-wire could be applied for cutting diamond substrates. However, few studies have been conducted on cutting diamond substrates with a diamond wire. A fixed diamond grain saw wire has been used to cut single crystal diamond plates, but the wire life and cutting length were short [17,18].

In this study, first, we improved the saw-wire-fixed grains. The grains were bonded using a reinforced metal solder to the wires by a metallic compound. Next, we investigated a method of producing a diamond saw-wire-fixed-grain using a metal solder in a vacuum chamber. Additionally, we developed a unique solder that contained a metallic compound [19,20,21]. Diamond grains could be bonded to the tungsten wires using this solder, which could be used for cutting extremely tough materials. Furthermore, we discussed the failure of the cutting mode when the micro-wire was less than approximately 100 μm in diameter [15]. Subsequently, we demonstrated that a fixed diamond grain-saw-wire could be used to control cutting of a single-crystal diamond plate. Finally, the cutting mechanism of the diamond plate using diamond grains, which is different from the conventional fixed grain saw wire-technique, was discussed [22].

## 2. Experimental Procedures

**Figure 1:**
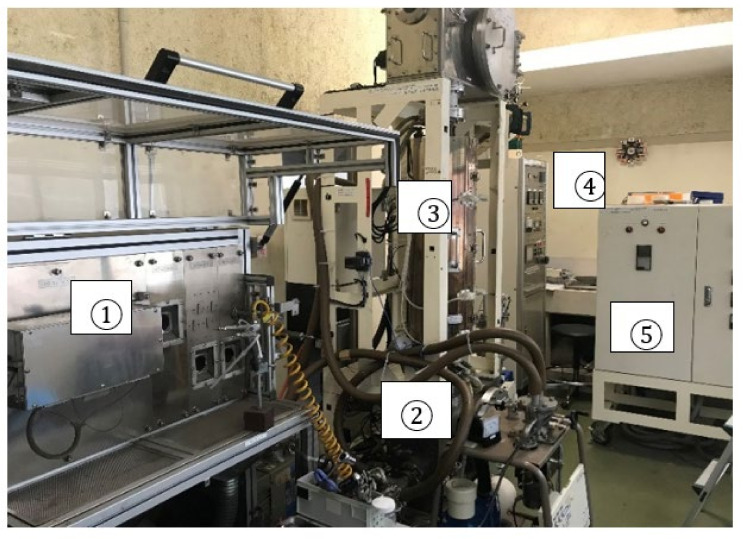
Production system of the diamond saw-wire employing a vacuum furnace. ① Gluing unit of diamond on tungsten wire using a gel. ② Vacuum pump. ③ Heating chamber. ④ Control panel unite. ⑤ Electrical power supply.

**Figure 2:**
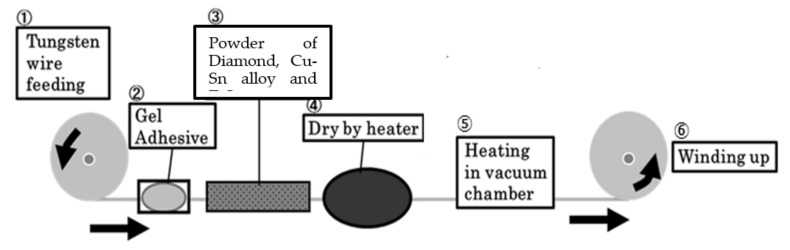
Schematic of continuous wire soldering process for pasting (**left**) and soldering reaction (**right**). ① Tungsten wire feeding, ② Gel Adhesive, ③ Powder of Diamond, Cu-Sn alloy and TiO_2_, ④ Dry by heater, ⑤ Heating in vacuum chamber, ⑥ Winding up.

A photograph of the entire system of the fixed diamond grain-saw-wire developed in this study is shown in Figure 1. The soldering conditions used in this study are presented in Table 1. The soldering process is schematically presented in Figure 2. The first step is the gluing of diamond (Figure 1①, Figure 2①–④), bronze alloy, and hydride grains onto a tungsten wire using a gel. The composition of the three powders used to form the mixture are listed in Table 2. The second step is the soldering process, which involves heating in a vacuum furnace (Figure 1③). Conventionally, stages 1 and 2 in the soldering process are conducted separately [23]. In this study, we improved the continuous wire soldering process. The material of the core wire used was tungsten, owing to its stable strength at a maximum heating temperature of 1173 K.

Connecting the atmosphere and the high vacuum was a technical challenge in this study. In our system, a 1.013 × 10^5^ Pa (1 atm) air atmosphere and 10^−3^ Pa vacuum atmosphere were connected. This was achieved via the three steps of the vacuum process described in Figure 1②. The wire was fed into the vacuum furnace, to melt the solder.

## 3. Results and Discussions

### 3.1. Production of the Metal Solder Fixed Diamond Grain-Saw-Wire

**Figure 3:**
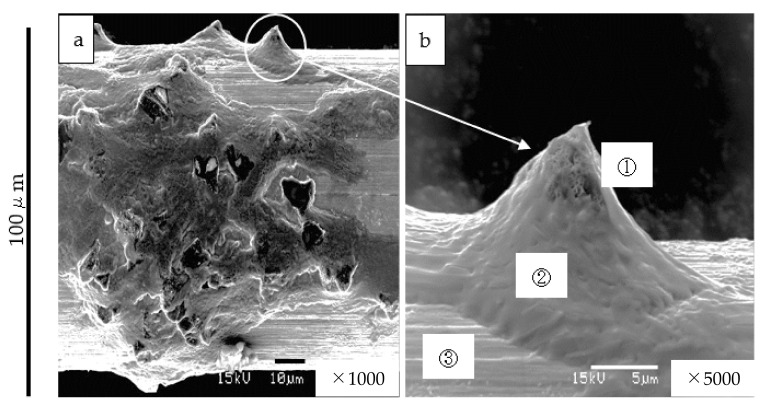
Fixed diamond grain saw wire by bronze (Cu-15% Sn) on tungsten-core wire. ① Diamond grain, ② bronze solder, ③ tungsten-core wire. (**a**) Whole appearance of the saw wire. (**b**) detail of the diamond grain fixed by the bronze solder.

The good wettability between the bronze solder, diamond grain, and tungsten core wire is shown in Figure 3. The tensile strength of the tungsten wire does not change after heating to 1173 K, because the recrystallization temperature of tungsten is 1473 K. We produced a fine-saw-wire of 100 µm diameter fixed diamond grain. This wire is one of the finest fixed grain-type diamond-saw-wires currently available in the industry.

The bonding strength (800 MPa) between the diamond and matrix core wire is close to the strength of the bronze solder, which was measured in a previous study [16,24]. Although, this strength is sufficient for the cutting tool, the grain distribution is not as uniform as that of industrial products. Therefore, the diamond-saw-wire produced in our laboratory is currently in the prototype stage.

### 3.2. Cutting Performance and Tool Life

**Figure 4:**
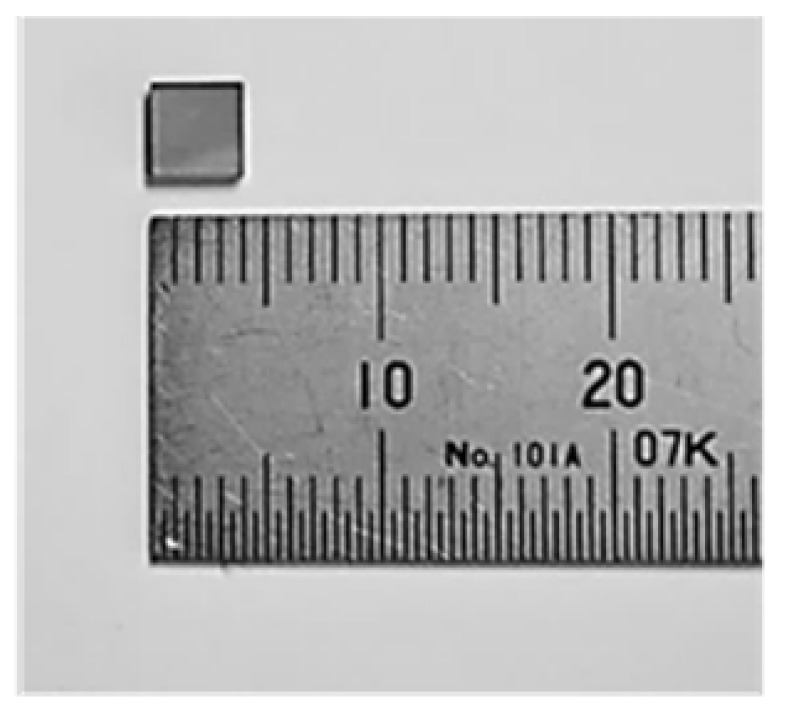
Diamond substrate with dimensions of 4 mm × 4 mm × 1 mm used for the cutting test.

**Figure 5:**
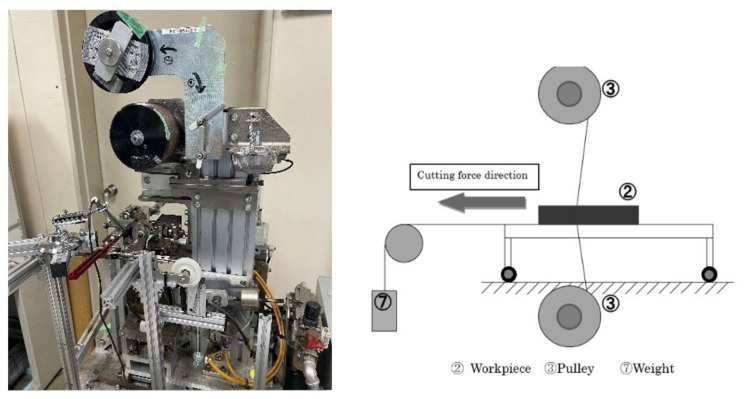
Cutting machine for diamond saw (**left**), and cutting diagram (**right**).

An industrially produced laser-cut single-crystal diamond substrate (4 mm × 4 mm × 1 mm), as shown in Figure 4, was used for the cutting test, using the fixed-grain-diamond-saw-wire, which was produced using the method described in the previous chapter. The cutting tests (Figure 5) were automatically performed using the conditions in Table 3.

**Figure 6:**
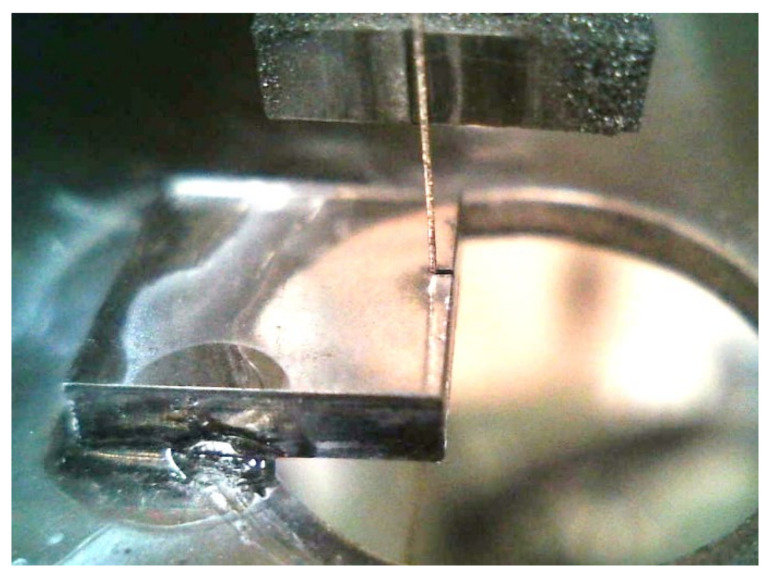
Cutting and manufacturing of a single-crystal diamond using a fixed diamond grain saw wire.

As shown in Figure 6, a saw wire of 8 m in length was attached to the end face (100) of the diamond substrate, and the wire reciprocating motion was automatically repeated so that the diamond could be slowly cut.

**Figure 7:**
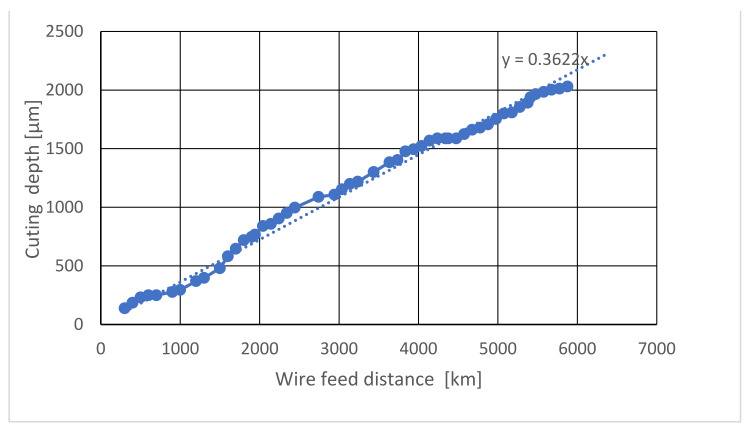
Linear relationship between the cutting depth and wire feed distance. Condition: cutting the diamond substrate using a saw wire with a thickness of 1 mm.

The cutting performance of this saw wire on the diamond substrate with a thickness of 1 mm is shown in Figure 7. A linear relationship was found between the cutting depth and wire feed distance. This relationship can be approximated using the formula y=0.3622x, where y $\left(\mu m\right)$ is the cutting depth, and x $\left(\mathrm{km}\right)$ is the wire feed distance. It was considered that the liner relationship of this equation is universal. However, the coefficient of 0.3622 depends on factors such as the coating density, solder type, wire speed, cutting load, and temperature.

The cutting speed ($v_{c}$), calculated using the formula y/x was 0.3622 µm/km, which is low and stable. The life of a saw-wire is the time elapsing between the start of its use and the breaking of the wire. As indicated in Figure 5, the life of the saw wire was longer than a 6000 km feed distance, while the length of the wire was 8 m, and it was repeatedly used in the cutting machine. The long life of the saw wire depends on the cutting force, as listed in Table 3. If the force is higher than 0.20 N, the life decreases significantly. As seen in Figure 7, the tested wire did not break, and half of the diamond grains remained.

### 3.3. Control Cutting

**Figure 8:**
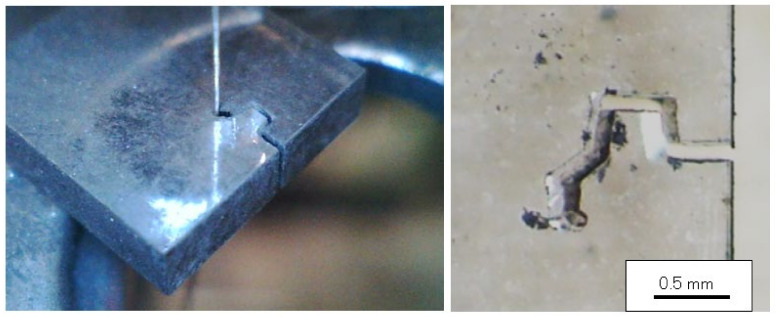
Control cutting of diamond plate with a thickness of 1 mm, using a fixed diamond grain saw-wire. The diameter of wire is 100 μm and the kerf width of the diamond substrate was 110 μm.

The control cutting of the diamond substrate using the saw-wire is represented in Figure 8. The diameter of the wire was measured as 100 μm and the kerf width of the diamond substrate was 110 μm. The six surfaces of the single-crystal diamond belonged to the (100) plane. The first cutting direction of the saw-wire was [100] and the length was 0.4 mm. The second direction was changed to [010], as shown in Figure 8. Therefore, the diamond was cut in different directions six times. During the cutting process, the cutting speed was constant. Therefore, it was possible to cut with a single saw-wire. We explored the possibility of the control-cutting of the diamond substrate using a saw-wire. However, the cutting speed was too slow to be used in industrial manufacturing. In future, it will be necessary to develop a method to increase the cutting speed.

## 4. Discussion of the Cutting Mechanism

When a diamond tool cuts materials with a low hardness value, its tip grain scratches and removes the surface of the work materials [22]. However, in the case of the diamond tool cutting diamond substrates, no scratch occurs because both the tool and substrate have the same hardness. Furthermore, an extremely low and stable cutting speed was observed when the diamond substrate was cut. Significantly, the fixed diamond-grain-type saw-wire developed in this study exhibited a long life.

**Figure 9:**
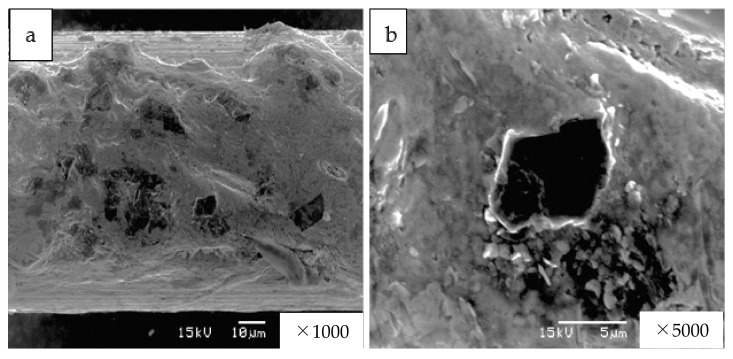
After 6000 km of running the saw-wire, (**a**) diamond grains existed, (**b**) worn and flattened surface of diamond grain was observed.

The diamond cutting mechanism in this study had a mild wear phenomenon, owing to the friction between the diamond surfaces. Figure 9 shows the worn and flattened diamond grain after 6000 km of running the saw-wire surface. These flattened diamond grains also maintained a constant cutting speed, as shown in Figure 7.

**Figure 10:**
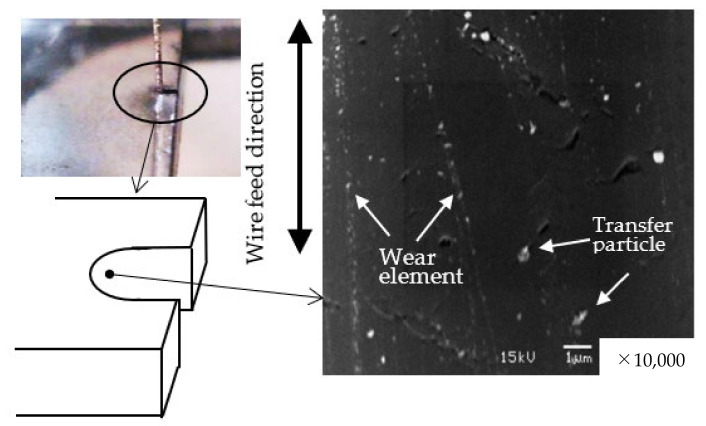
Smooth surface of diamond cutting tip in contact with the saw wire.

As shown in Figure 10, no mechanical scratches were noticed, but wear elements and transfer of particles were observed at the diamond surface cut by the saw-wire.

**Figure 11:**
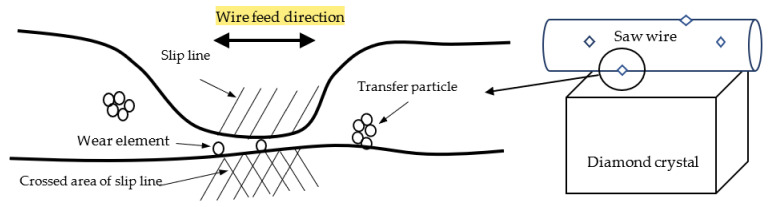
Schematic of the diamond cutting mechanism.

The diamond cutting mechanism applied in this study can be explained using the standard wear mechanism [25], as shown in Figure 11. It was hypothesized that the diamond cutting phenomenon would be consistent with the wear mechanism. Wear of the diamonds occurred in the following five stages:

(i) Atomic-level diamond surface asperities came into contact with each other.
(ii) Slip lines occurred on the diamond surface, owing to the contact stress.
(iii) The crossed area of slip lines generated the diamond wear elements.
(iv) The wear elements grew with transferred particles.
(v) Transferred particles were eliminated from the diamond surface.

The size of the wear element was smaller than 100 nm, while the transferred particles were approximately 300 nm in size and, were observed as white particles (Figure 10). Wear volume *V* is expressed using Holm’s equation, as follows [26]:

(1) $$V=\frac{Z\times P\times \ell }{p_{m}},$$

where *V* is the diamond cutting volume; *P* (N) is the applied cutting load; $p_{m}$ (Pa) is the yield stress of the diamond; ℓ (m) is the wire feed distance, and *Z* (mm^3^/m) is the wear coefficient. We calculated the *Z* of diamond for diamond saw-wire cutting, using the test condition parameters listed in Table 4.

We substituted the values listed in Table 3 into Equation (1) and calculated the wear coefficient Z as 1.811 × 10^−2^. Therefore, Equation (1) can be rewritten as:

(2) $$V=1.811\times 10^{-2}\frac{P}{p_{m}}\times \ell .\;\;\;\;\;\;\;\left[{\mathrm{mm}}^{3}\right]\;$$

Equation (2) can be used to calculate the value of *V* using the values of *P* and ℓ. It was reasoned that the value of Z could be affected by various factors, such as the diamond grain size, grain density, cutting temperature, cutting liquid type, or additional element in the counter surface. In future studies, the effects of such factors will be investigated.

The application of the method investigated in this study is limited. Moreover, the proposed method is unsuitable for the mass production process because this method has an extremely slow cutting speed; the kerf is 110 μm and the cutting length is limited to 2000 μm.

Based on the wear equation of diamond, the cutting volume exhibits a linear relationship to the applied load and wire feed distance. The contact stress of the grain tip reduces as the shape of the diamond grain tip changes from keen to fiat (Figure 3 and Figure 9). Accordingly, the number of scratches and degree of cutting of the workpiece by the grain tip reduce. However, the diamond cutting by the diamond grain is based on the wear phenomenon that is presented by Equation (2). This is because the stress changes but not the total load *P*. Therefore, the wear volume of the diamond substrate does not change. It can be concluded that the mild wear amount *V* is not related to the stress at the grain tip but is related to the applied cutting load *P*. In future, studies to improve the cutting speed by optimizing the mechanical conditions of cutting load, wire speed, temperature, and additional processing promotion material will be conducted.

## 5. Conclusions

In this study, we investigated the possibility of cutting a single crystal diamond substrate using a fixed-grain-diamond-saw-wire and the control-cutting of the single-crystal diamond substrate using the saw-wire. The results are summarized as follows:

(1) A fixed grain diamond-saw-wire was successfully produced in a high vacuum furnace, using bronze solder and by adding titanium hydride powder.
(2) The relationship between the cutting length and wire-feed distance was linear and can be represented using an approximate formula y=0.3622x, where y is the cutting depth and x is the wire feed distance. The coefficient of 0.3622 depends on the test conditions.
(3) The life of the saw-wire was longer than that of the 6000 km feed distance, which was tested by reciprocating an 8-m short wire at a speed, tension, and cutting force of 150 m/min, 1 N, and 0.2 N, respectively.
(4) A single crystal diamond substrate could be control-cut along the designed line using the fixed diamond-saw-wire
(5) The cutting speed of the diamond cutting was maintained constant at 0.36 μm/km (cutting length/feed length of the saw wire).
(6) The diamond cutting mechanism in this study can be explained by considering the mild wear phenomenon, owing to the friction between the diamond and diamond surfaces.

## Figures and Tables

**Table 1:** Soldering condition.

Conditions	Unit	Value
Tungstain core wire diameter	(μm)	80
Brazing filler metal		Bronze (Cu-15% Sn)
Melting temperature of bronze T_melt_ *	(K)	1071
Diamond grain diameter	(μm)	3.0
Brazing atmosphere		Vacuum (10^−2^–10^−3^ Pa)
Redox material		Titanium hydride (TiH_2_)
Temperature of heating chamber	(K)	1173
Wire production velocity	(mm/s)	83.3
Tension load	(N)	1

* T_melt_: melting start temperature.

**Table 2:** Composition of solder including the diamond grain (mass%).

Bronze Cu-15% Sn	Titanium Hydride TiH_2_	Diamond Grain φ3 μm
86.2	8.6	5.2

**Table 3:** Cutting conditions for this study.

Tension (N)	Cutting Force (N)	Wire Speed (m/s)	Wire Length (m)	Cutting Environment
1	0.20	2.5	8	Dry cutting

**Table 4:** Cutting condition parameters.

Conditions	Unit	Value
*V*	(mm^3^)	3.622 × 10^−5^
P	(N)	0.2
p_m_	(GPa)	90–100
*ℓ*	(m)	1.0 × 10^3^
